# Semaglutide exerts neuroprotective effects by blocking the interleukin-17/NOD-like receptor family pyrin domain containing 3-mediated neuroinflammation pathway after traumatic brain injury

**DOI:** 10.4103/NRR.NRR-D-25-00990

**Published:** 2026-01-27

**Authors:** Bin Zhang, Lu Kong, Yumei Wang, Wei He, Mengshi Yang, Xueling Zhang, Xiyu Chen, Yaxuan Zhang, Baiyun Liu, Miao Bai, Guangzhi Shi

**Affiliations:** 1 Department of Critical Care Medicine, Beijing Tiantan Hospital, Capital Medical University, Beijing, China; 2 Department of Neurosurgery, Qingdao Municipal Hospital, Qingdao, Shandong Province, China; 3 Department of Neurosurgery, Qilu Hospital of Shandong University (Qingdao), Qingdao, Shandong Province, China; 4 Department of Neurosurgery, Beijing Tiantan Hospital, Capital Medical University, Beijing, China; 5 Department of Neurology, The First Hospital of Tsinghua University, Beijing, China

**Keywords:** apoptosis, blood–brain barrier, brain edema, glucagon-like peptide-1 receptor, interleukin-17, neuroinflammation, neuronal survival, secondary brain injury, semaglutide, traumatic brain injury

## Abstract

Semaglutide, a long-acting glucagon-like peptide-1 receptor agonist, exhibits significant neuroprotective effects in stroke and neurodegenerative diseases. However, the anti-inflammatory and barrier-protective effects of semaglutide and the mechanisms underlying its effects following traumatic brain injury remain unclear. In this study, we used mice to establish a model of controlled cortical impact injury to investigate the roles and effects of semaglutide on neuroinflammation, the integrity and permeability of the blood–brain barrier, brain edema, and the recovery of neurological function after traumatic brain injury. Our results showed that semaglutide treatment alleviated interleukin-17/NOD-like receptor pyrin domain-containing 3-induced neuroinflammation and upregulated the expression of tight junction proteins, thereby reducing brain leakage and edema while promoting the recovery of neurological function. Furthermore, transmission electron microscopy assessments demonstrated that semaglutide maintained the ultrastructure of the blood–brain barrier, strengthening the tight junctions between endothelial cell membranes. Mechanistically, semaglutide alleviated inflammatory conditions by interrupting the positive-feedback loop of inflammation in peripheral and innate immune cells induced by interleukin-17/NOD-like receptor pyrin domain-containing 3. Collectively, our findings reveal the dual anti-inflammatory and neuroprotective roles of semaglutide, providing important preclinical evidence for its clinical application in the acute phase of traumatic brain injury.

## Graphical abstract

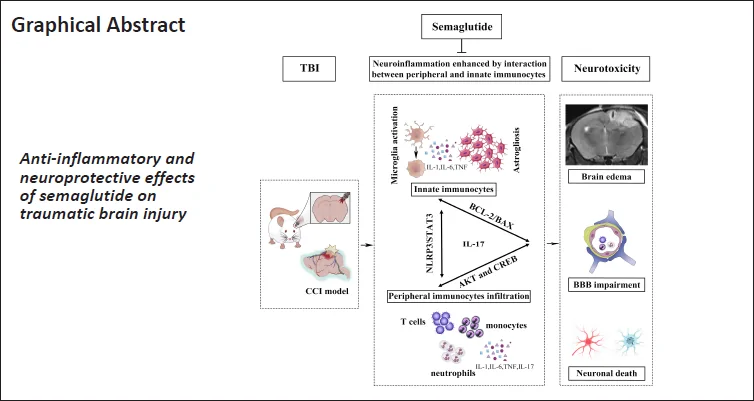

## Introduction

Traumatic brain injury (TBI) is a severe condition affecting more than 50 million individuals annually and remains the leading cause of death and long-term disability in children and young adults worldwide, placing an enormous burden on global healthcare systems (Maas et al., 2022). In addition to the primary mechanical brain injury immediately after the incident, TBI also leads to ongoing secondary damage, which can be attributed to complex pathophysiological cascades involving inflammation, brain edema, and increased intracranial pressure. These processes persist into the sub-acute phase and can progress into the chronic phase even after surgical interventions (Wilson et al., 2017).

Despite advancements in neuro-monitoring techniques and surgical intervention methods (such as decompressive craniectomy), effective drug strategies targeting these interrelated secondary processes are lacking. This gap severely hinders improvements in the prognosis of TBI and highlights the urgent need to develop therapeutic strategies that can both alleviate acute injuries and support neural repair and regeneration pathways, thereby allowing long-term functional recovery.

**Figure:**
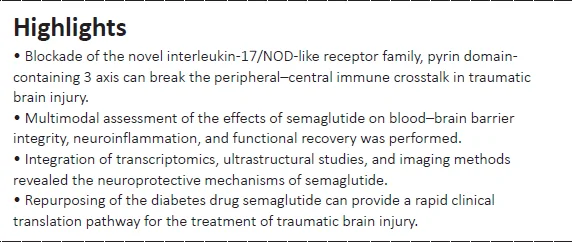

Decades of preclinical and clinical investigations into anti-inflammatory and edema-modulating agents have yielded limited translational success for the treatment of brain edema after TBI (Freire et al., 2023; Van et al., 2023; Zima et al., 2024). For example, corticosteroids, once considered the standard agents for managing cerebral edema, show minimal efficacy in severe TBI and are associated with systemic complications such as infection, metabolic derangements, and increased mortality (Gobiet et al., 1976; Edwards et al., 2005). Similarly, while osmotic agents such as mannitol provide transient relief from intracranial pressure, they cannot disrupt the underlying inflammatory cascades driving secondary injury (Rowland et al., 2020; Van et al., 2023; Bernhardt et al., 2024). This gap between experimental promise and clinical reality underscores the urgent need for therapies that directly target neuroinflammatory pathways with high specificity.

Glucagon-like peptide-1 receptor agonists (GLP-1RAs), which were originally developed for type 2 diabetes, have recently emerged as promising neuroprotective candidates. GLP-1Rs are densely expressed in TBI-vulnerable brain regions (e.g., hippocampus and cerebral cortex) and are localized to neurons, astrocytes, and microglia, allowing dual modulation of neural survival and immune responses (Kabahizi et al., 2022; Hölscher, 2024). Preclinical studies have shown that short-acting GLP-1RAs (e.g., liraglutide, exendin-4) suppress neuroinflammation, enhance blood–brain barrier (BBB) integrity, and promote synaptic plasticity in models of Alzheimer’s disease (AD), Parkinson’s disease (PD), and ischemic stroke (Salameh et al., 2020; Zhang et al., 2023). Semaglutide (SEMA), a long-acting GLP-1RA with a 1-week plasma half-life, has been shown to offer distinct advantages for neurological conditions requiring sustained action due to its prolonged pharmacokinetics (Maskery et al., 2021; Nizari et al., 2021; Hong et al., 2024; Hölscher, 2024). However, its therapeutic potential in TBI, specifically in relation to neuroinflammation, acute-phase cerebral edema, BBB dysfunction, and the downstream signaling pathways remains uncharacterized.

Against this backdrop, we hypothesized that SEMA could mitigate post-TBI inflammation and cerebral edema through dual mechanisms: suppressing neuroinflammatory responses and preserving the structural integrity of the BBB. This study aimed to investigate whether SEMA administration attenuates secondary brain injury in a murine controlled cortical impact model by (1) quantifying edema using dry/wet analysis and magnetic resonance imaging (MRI); (2) assessing BBB integrity and permeability; (3) profiling the effects of SEMA on neuroinflammatory cytokines and immune cells by multiplex analysis (RNA sequencing [RNA-Seq], reverse transcription [RT]-quantitative polymerase chain reaction [qPCR], western blot and immunofluorescence analyses, and enzyme-linked immunosorbent assay [ELISA]); and (4) evaluating neurological functions. By integrating the findings of these multimodal approaches, we sought to demonstrate the efficacy of a long-acting GLP-1RA in acute TBI, address the unmet need for sustained pharmacological intervention, establish a mechanistic link between SEMA-mediated GLP-1R activation, BBB preservation, and neuroinflammation suppression, and accelerate translational research, laying the groundwork for repurposing SEMA as a clinically viable therapeutic agent for TBI.

## Methods

### Animals

All experimental procedures were authorized by the Institutional Animal Care and Use Committee of Beijing Neurosurgical Institute (approval No. 202204008; approval date: March 3, 2022). Adult male C57BL/6J mice (*n* = 210; age: 10–12 weeks; weight: 25–30 g) were purchased from Beijing Vital River Experimental Animals Technology Ltd. and utilized in this study (Animal license number: SYXK(jing)2024-0003. All mice were housed individually, provided with unrestricted access to food and water, and habituated to the animal facility conditions for 7 days before the beginning of the experiments. The environment was maintained at a controlled temperature of 22°C and humidity of 60%. All experiments were designed and reported according to the Animal Research: Reporting of *In Vivo* Experiments (ARRIVE) guidelines (Percie du Sert et al., 2020) and the National Institutes of Health Guide for the Care and Use of Laboratory Animals (8^th^ ed., National Research Council, 2011).

### Controlled cortical impact model

The controlled cortical impact (CCI) brain injury model was established using a PCI3000 PinPoint Precision Cortical Impactor (Hatteras Instruments, Cary, NC, USA), as described previously (Zhang et al., 2020). Mice underwent isoflurane inhalation for anesthesia and were fixed on a stereotaxic apparatus (RWD Life Science Co., Shenzhen, Guangdong, China). After making an incision on the scalp and exposing the skull, a craniotomy (approximately 4 mm in diameter) was performed at the center of the right parietal bone, and the underlying dura mater was kept intact. A severe TBI model was then established using a 3-mm circular impact tip and the impact parameters reported previously (velocity: 3.5 m/s; compression time: 400 ms; depth: 2 mm) (Zhang et al., 2025). Throughout the surgical procedure, a thermal pad was used to maintain the mouse body temperature at approximately 37.0 ± 0.5°C. Transient respiratory depression occurred post-impact, and the presence of cortical edema, hemorrhage, or necrosis in the impact-injured area observed postoperatively was deemed indicative of successful establishment of the CCI model.

### Grouping, treatments, and tissue preparation

Mice were randomly assigned to three groups on the basis of the CCI model and SEMA treatments: 1) Sham + sodium chloride group (Sham, *n* = 62); 2) CCI + sodium chloride group (CCI, *n* = 62); 3) CCI + SEMA group (SEMA, *n* = 62). The SEMA injection (1.34 mg/mL 1.5 mL Novo Nordisk, China) was diluted by 0.9% sodium chloride into a 0.02-mg/mL working liquid and then stored in refrigerator at 4°C away from light. The detection time points were post-injury days 3 and 7. Therefore, the SEMA working liquid was injected intraperitoneally at a dose of 0.25 mg/kg on post-injury days 1 and 5 (Poupon-Bejuit et al., 2024). In the absence of standardized dosages or administration cycles for animal studies on the neuroprotective effects of SEMA, the dosage and administration interval selected in this study were referenced from the majority of similar preclinical studies to ensure methodological rationality.

### Immunofluorescence staining

For histological analysis, mice (*n* = 6 per group) were euthanized on post-injury days 3 and 7. Briefly, mice were anesthetized using 2% isoflurane (RWD Life Science Co.,) and 100% oxygen at a delivery rate of 1 L/min. After transcardiac perfusion with 0.9% saline and 4% paraformaldehyde, their brains were immediately removed, fixed, and embedded. The fixed tissues were sectioned into 5-μm coronal sections. Paraffin-embedded brain slices were dewaxed and rehydrated with xylene and ethanol for immunofluorescence staining, as described previously (Yang et al., 2025). Briefly, brain slices were stained singly (glial fibrillary acidic protein [GFAP], post-injury day 3) or doubly (CD16/32 and Iba-1, CD34/platelet-derived growth factor receptor beta [PDGFRβ] post-injury day 3) for immunohistochemical evaluation. In brief, the brain slices underwent incubation with the primary antibodies overnight at 4°C. After incubation with the primary antibodies, the sections were washed three times with phosphate-buffered saline (PBS) and incubated in the following fluorescent secondary antibodies at 25 ± 2°C for 2 hours: Alexa Fluor 647-conjugated donkey anti-rabbit IgG (1:500, Abcam, Cambridge, UK, Cat# ab150080, RRID: AB_2650602) and Alexa Fluor 488-conjugated goat anti-mouse IgG (1:500, Abcam, Cat# ab150113, RRID: AB_2576208). Nuclei were counterstained with 4′,6-diamidino-2-phenylindole (DAPI) (Cat# D9542, Sigma-Aldrich, St. Louis, MO, USA) for 10 minutes to visualize cell populations. The antibodies used are listed as follows: rabbit polyclonal anti-GFAP (1:5000, Abcam, Cat# ab7260, RRID: AB_305808); rabbit monoclonal anti-CD16/32 (1:500, Abcam, Cat# ab223200); rabbit monoclonal anti-ionized calcium-binding adapter molecule 1 (Iba-1) (1:200, Abcam, Cat# ab178846, RRID: AB_2636859); rabbit monoclonal anti-CD34 (1:200, Abcam, Cat# ab81289, RRID: AB_1640331); and mouse monoclonal anti-PDGFRβ (1:500, Abcam, Cat# ab69506, RRID: AB_1269704). The number of microglia, endotheliocytes, and pericytes in the ipsilateral cortex and hippocampus were quantified. Digital images of the entire brain sections were captured using a MIDI FL system (Pannoramic P250 FLSAH, 3D Histech, Hungary). Three equally sized, non-overlapping zones in the perilesional cortex or the ipsilateral hippocampus were acquired for cell counts. Data were presented as the number of positive cells per mm^2^.

### Nissl staining

Nissl staining was conducted on post-injury day 7 (*n* = 6 per group) in accordance with the manufacturer’s protocol for the staining kit (Cat# G1032; Servicebio, China), with paraffin-embedded brain sections (3–5 μm thickness) prepared as described previously (Yang et al., 2025). Briefly, sections were first baked at 60°C for 2 hours to enhance tissue adhesion, then dewaxed by sequential immersion in xylene I and xylene II for 10 minutes each, followed by rehydration through a graded ethanol series, and final rinsing in distilled water for 2 minutes. The rehydrated sections were incubated in pre-warmed (25–28°C) toluidine blue-based Nissl staining solution for 5 minutes and differentiated in 0.5% hydrochloric acid-ethanol for 30 seconds to reduce background staining, followed by immediate rinsing with distilled water for 1 minute to terminate differentiation. Subsequent dehydration was performed using ascending ethanol concentrations and xylene, and the sections were mounted with neutral balsam to preserve tissue morphology. For microscopic observation, the sections were examined under an optical microscope, with neurons identified by their intact cellular morphology, distinct round nuclei, and characteristic distribution of dark blue granular Nissl bodies in the cytoplasm. Digital images of the entire brain sections were captured using a MIDI FL system (Pannoramic P250 FLSAH; 3D Histech, Hungary). Three equally sized, non-overlapping zones in the perilesional cortex or the ipsilateral hippocampus were acquired for cell counts. Data are presented as the number of cortical neurons per mm^2^ and the number of hippocampal neurons per mm^2^.

### Quantification of apoptotic cells

Apoptotic cell detection was performed on post-injury day 3 (*n* = 6 per group) using the terminal deoxynucleotidyl transferase dUTP nick-end labeling (TUNEL) detection kit (Cat# 11684817910, Roche, Indianapolis, IN, USA) in accordance with the manufacturer’s protocol. Briefly, sections were processed for dewaxing and rehydration, and then treated with proteinase K to achieve antigen retrieval. A TUNEL reaction mixture (consisting of terminal deoxynucleotidyl transferase and dUTP) was applied to the sections, followed by a 2-hour incubation at 37°C. After two rounds of PBS washing, the sections were incubated with an anti-fluorescein antibody conjugated with peroxidase (Converter-POD) for 1 hour at 37°C, allowing the visualization of labeled DNA breaks. Nuclei were counterstained with DAPI to complete the process. The number of apoptotic cells in the ipsilateral cortex and hippocampus was quantified. Digital images of the entire brain sections were captured using a MIDI FL system (Pannoramic P250 FLSAH, 3D Histech, Hungary). Three equally sized, non-overlapping zones in the perilesional cortex or the ipsilateral hippocampus were acquired for cell counts. Data were presented as the number of positive cells per mm^2^ and the number of the hippocampal TUNEL^+^ cells.

### RNA sequencing and data analysis

To investigate the molecular mechanisms driving TBI-induced secondary brain injury, transcriptomic profiling was conducted by next-generation sequencing on post-injury day 3 (*n* = 3 per group), in accordance with a previously reported method (Yang et al., 2025). Total RNA was isolated from the ipsilateral perilesional cortex of mouse brain tissues using TRIzol reagent (Invitrogen, Carlsbad, CA, USA). On the basis of assessments of RNA purity and integrity by a NanoDrop 2000 spectrophotometer (Thermo Fisher Scientific, Waltham, MA, USA), poly(A) RNA was isolated, fragmented, and reverse-transcribed into a complementary DNA (cDNA) library with the mRNA-Seq sample preparation kit (Illumina, San Diego, CA, USA). Paired-end sequencing was performed on an Illumina NovaSeq 6000 platform. After filtering raw reads, high-quality reads were mapped to the mouse reference genome using HISAT2 (http://www.ccb.jhu.edu/software/hisat/index.shtml), a tool that integrates splice junction information to enable accurate alignment in complex transcriptomes. The mapped reads were then assembled and quantified with StringTie (v1.3.0; http://ccb.jhu.edu/software/stringtie/), which calculates gene and transcript expression levels through integration of genomic annotation and novel transcript identification. Differential gene expression analysis was performed using DESeq2 (v1.34.0), where genes with |log_2_(fold change)| > 1 and a false discovery rate (FDR) < 0.05 were defined as significantly differentially expressed genes (DEGs). To identify differentially expressed transcription factors among the DEGs at the mRNA level, a list of mouse transcription factors was downloaded from the Animal Transcription Factor Database (AnimalTFDB; http://bioinfo.life.hust.edu.cn/AnimalTFDB/#!/) and utilized for screening.

### Kyoto encyclopedia of genes and genomes pathway and gene ontology enrichment analyses

Functional enrichment analyses based on Kyoto Encyclopedia of Genes and Genomes (KEGG) pathways and gene ontology (GO) annotations were performed using Metascape (Zhou et al., 2019), a comprehensive platform integrating multiple biological databases. To identify significantly enriched terms while controlling for multiple testing bias, the Benjamini–Hochberg procedure was used to calculate FDRs. Terms with an adjusted FDR < 0.05 were considered statistically significant, indicating enrichment in biological processes, molecular functions, or cellular components relevant to the studied phenotype.

### RNA extraction and quantitative reverse transcription-polymerase chain reaction

RT-qPCR was performed to determine the expression levels of inflammatory factors in the perilesional cortical tissue at post-injury day 3 (*n* = 5 per group). Using a previously documented protocol (Yang et al., 2025), total RNA was isolated from the ipsilateral cortical tissue by TRIzol reagent (Invitrogen, Carlsbad, CA, USA). Subsequently, 1 μg of total RNA was reverse-transcribed into cDNA with the Prime-Script RT Reagent Kit (Thermo Fisher Scientific, Waltham, MA, USA). Next, the synthesized cDNA was subjected to amplification via RT-qPCR using a Quant Studio^TM^ 5 System, with SYBRTM Select Master Mix (Life Technologies, Carlsbad, CA, USA) added as the reaction reagent. β-Actin served as the housekeeping gene to normalize the expression levels of the target genes. mRNA expression levels were finally expressed as fold changes relative to the sham control group. The primer sequences for the target genes are provided in **Table 1**.

**Table 1:** Primer sequences used in quantitative reverse transcription-polymerase chain reaction

Gene	Sequence (5'–3')	Product size (bp)
*Il-17a*	F: TTT AAC TCC CTT GGC GCA AAA	21
	R: CTT TCC CTC CGC ATT GAC AC	20
*Il-17f*	F: TGC TAC TGT TGA TGT TGG GAC	21
	R: AAT GCC CTG GTT TTG GTT GAA	21
*NLRP3*	F: ATT ACC CGC CCG AGA AAG G	19
	R: TCG CAG CAA AGA TCC ACA CAG	21
*ASC*	F: CTT GTC AGG GGA TGA ACT CAA AA	23
	R: GCC ATA CGA CTC CAG ATA GTA GC	23
*Caspase1*	F: ACA AGG CAC GGG ACC TAT G	19
	R: TCC CAG TCA GTC CTG GAA ATG	21
*IL-1β*	F: GCA ACT GTT CCT GAA CTC AAC T	22
	R: ATC TTT TGG GGT CCG TCA ACT	21
*TNF-α*	F: CAG GCG GTG CCT ATG TCT C	19
	R: CGA TCA CCC CGA AGT TCA GTA G	22
*IL-6*	F: CTG CAA GAG ACT TCC ATC CAG	21
	R: AGT GGT ATA GAC AGG TCT GTT GG	23
*β-actin*	F: GTG ACG TTG ACA TCC GTA AAG A	22
	R: GCC GGA CTC ATC GTA CTC C	19

ASC: Apoptosis-associated speck-like protein containing a caspase recruitment domain; F: forward; IL-1β: interleukin 1β; IL-17a: interleukin 17a; IL-17f: interleukin 17f; IL-6: interleukin 6; NLRP3: NOD-like receptor pyrin domain-containing 3; R: reverse; TNF-α: tumor necrosis factor-alpha.

### Enzyme-linked immunosorbent assay

The levels of inflammatory cytokines, including tumor necrosis factor (TNF)-α, interleukin (IL)-6, and IL-1β, were evaluated in the ipsilateral hemisphere. Tissue samples from the ipsilateral hemisphere were collected at post-injury day 3 (*n* = 6 per group). After homogenization, supernatants were obtained and used for further analysis. Cytokine levels were measured using specific enzyme-linked ELISA kits (Mouse IL-6: Cat# ab222503; Mouse IL-1β: Cat# ab197742; Mouse TNF-α: Cat# ab208348, Abcam, Cambridge, UK) in accordance with the manufacturer’s instructions.

### Determination of brain water content

For the assessment of brain water content, the wet and dry weights of brain tissue were measured on post-injury day 3 (*n* = 6 per group). Mice brains were removed without prior transcardial perfusion. The injured cerebral hemisphere was then carefully dissected, and its wet weight was measured immediately. Next, the dissected tissue was placed in an 80°C oven for 72 hours of drying, until a stable dry weight was obtained. Brain water content was calculated using the following formula: Brain water content (%) = [(wet weight – dry weight)/wet weight] × 100%.

### Evans blue extravasation

To assess BBB permeability, Evans blue (EB) extravasation into the injured hemisphere was quantified on post-injury day 3 (*n* = 6 per group). Mice were injected with a 4% EB solution (3 mL/kg; Cat# E2129, Sigma-Aldrich) through the tail vein. After a 4-hour period, the animals were anesthetized and transcardially perfused with PBS to remove circulating EB. The injured cerebral hemisphere was then dissected, weighed, and homogenized in formamide at 72°C for 3 days to ensure complete extraction of EB. After centrifugation, the supernatant was harvested, and the absorbance of the supernatant was determined at 620 nm with a spectrophotometer (Bio-Rad, Hercules, CA, USA).

### Magnetic resonance imaging

An animal magnetic resonance (MR) scanner (Bruker 7.0 T, Germany) was employed to assess the volume of brain edema on post-injury day 3 (*n* = 6 per group). As described previously (Zhang et al., 2025), all animals were fully anesthetized with pentobarbital. Each group underwent axial T1/T2/apparent diffusion coefficient imaging with the following parameters: field of view, 35 mm × 35 mm; slice thickness, 1 mm. The MR images were read and reviewed using RadiAnt DICOM viewer (version 4.6.9, Medixant, Poznan, Poland) and annotated by radiologists.

### Western blot analysis

Western blot analysis was performed to quantify the expression levels of the signaling pathway proteins on post-injury day 3 (*n* = 6 per group). To isolate the proteins, the ipsilateral hemisphere was homogenized using a motor-driven homogenizer. Homogenates were then centrifuged to pellet cellular debris at 15,000 × *g* for 10 minutes at 4°C, and the resulting supernatants were collected for further analysis. Protein concentrations were measured by a bicinchoninic acid (BCA) assay (Cat# G2026, Servicebio, China), and equal amounts of protein (30 μg) were separated by sodium dodecyl sulfate-polyacrylamide gel electrophoresis (Cat# B1012, Applygen, Beijing, China). After electrophoresis, proteins were electroblotted onto methanol-activated polyvinylidene fluoride membranes (Millipore, Boston, MA, USA) at a constant current of 400 mA for 90 minutes. Membranes were blocked overnight at 4°C in Tris-buffered saline with Tween 20 containing 5% non-fat dry milk on a rocking platform to avoid non-specific antibody binding. After blocking, the membranes were incubated with primary antibodies overnight at 4°C. Subsequently, the membranes were washed three times with Tris-buffered saline with Tween 20 (15 minutes per wash) and then incubated with matching secondary antibodies (anti-mouse IgG antibody, Abcam, Cat# ab6728, RRID: AB_955440; anti-rabbit IgG antibody, Cat# ab205718, RRID: AB_2819160) diluted at 1:5000 in blocking buffer for 60 minutes at room temperature. Protein bands were visualized using an enhanced chemiluminescence detection system: equal volumes of enhanced chemiluminescence reagents 1 and 2 were mixed, and bands were imaged using the Bio Spectrum 500 Imaging System (UVP, Upland, CA, USA). ImageJ software (v1.49, National Institutes of Health, Bethesda, MD, USA) was used to quantify relative band density, which was normalized to that of glyceraldehyde 3-phosphate dehydrogenase. For each sample, the percentage of expression relative to sham controls was calculated.

The primary antibodies used were as follows: rabbit polyclonal anti-IL-17A (1:1000, Cat# ab79056, RRID: AB_1603584, Abcam); rabbit monoclonal anti-IL-17F (1:1000, Cat# ab187059, Abcam); rabbit monoclonal anti-NOD-like receptor pyrin domain-containing 3 (NLRP3) (1:1000, Cat# ab263899, RRID: AB_2889890, Abcam); rabbit recombinant multiclonal anti-apoptosis-associated speck-like protein containing a caspase recruitment domain (ASC) (1:1000, Cat# ab309497, RRID: AB_3669034, Abcam); rabbit monoclonal anti-caspase-1 (1:1000, Cat# 2225, Cell Signaling Technology, Danvers, MA, USA); rabbit monoclonal anti-signal transducer and activator of transcription (STAT) 3 (1:1000, Cat# ab68153, RRID: AB_2889877, Abcam); rabbit monoclonal anti-p-STAT3 (Y705, 1:1000, Cat# ab76315, RRID: AB_1658549, Abcam); rabbit polyclonal anti-protein kinase B (AKT) (1:500; Cat# ab8805, RRID: AB_306791, Abcam), rabbit monoclonal anti-p-AKT (ser 473, 1:5000; Cat# ab81283, RRID: AB_2224551, Abcam); rabbit monoclonal anti-cAMP-response element binding protein (CREB) (1:1000; Cat# ab32515, Abcam), rabbit polyclonal anti-p-CREB (ser133, 1:5000; Cat# ab194687, RRID: AB_2749855, Abcam); rabbit polyclonal anti-B-cell lymphoma 2 (Bcl-2) (1:1000; Cat# ab59348, RRID: AB_2064155, Abcam); rabbit monoclonal anti-Bcl-2 associated X protein (Bax) (1:5000; Cat# ab32503, RRID: AB_725631, Abcam); rabbit monoclonal anti-occludin (1:1,000, Cat# ab224526, Abcam, UK); rabbit monoclonal anti-claudin-5 (1:1000, Cat# ab131259, RRID: AB_11157940, Abcam); rabbit monoclonal anti-zonula occludens (ZO)-1 (1:1000, Cat# ab276131, RRID: AB_3083081, Abcam); mouse monoclonal anti-glyceraldehyde-3-phosphate dehydrogenase (1:5000, Cat# ab8245, RRID: AB_2107448, Abcam).

### Transmission electron microscopy

On post-injury day 3, mice were transcardially perfused with 0.1M PBS (pH 7.4) followed by a fixative solution containing 4% paraformaldehyde and 2.5% glutaraldehyde in 0.1 M PBS (*n* = 6). As described previously (Zhang et al., 2025), ipsilateral cortical and hippocampal tissues adjacent to the injury core were dissected and post-fixed in the same fixative for 24 hours at 4°C. The samples were then trimmed into 1-mm³ blocks and rinsed three times (10 minutes each) in 0.1 M PBS. Tissue blocks were dehydrated through a graded ethanol series (50%, 70%, 90%, 100%) and embedded in Epon 812 resin. Ultrathin sections (70–90 nm) were cut using a diamond knife on a ultramicrotome (UC7; Leica, Wetzlar, Germany) and mounted on copper grids. Sections were stained with 2% uranyl acetate (15 minutes) and lead citrate (5 minutes) to enhance contrast. The ultrastructure of the BBB, including endothelial cell tight junctions, basement membrane integrity, and perivascular astrocyte endfeet, was examined using a transmission electron microscope (HT7800, Hitachi, Japan) operated at 80 kV. Images were captured at 10,000× to 30,000× magnification, focusing on regions exhibiting tight junctions and astrocyte endfoot swelling.

### Behavioral tests

#### Morris water maze

The Morris water maze (MWM) test was performed to assess spatial learning and reference memory in mice (*n* = 6 per group) starting from post-injury day 7, with modifications based on a previously described protocol (Zhang et al., 2020). The apparatus consisted of a circular pool (120 cm in diameter, 50 cm in height) filled with opaque water (22 ± 1°C). A hidden escape platform (10 cm in diameter) was submerged 1 cm below the water surface in the target quadrant (northeast quadrant) throughout the training phase. Training sessions were conducted daily for 5 consecutive days (days 7–11 post-CCI), with four trials per mouse per day and an inter-trial interval of 30 minutes. For each trial, the mouse was gently placed into the water facing the pool wall from one of four random starting positions (northwest, southwest, southeast, or northeast) and allowed a maximum of 120 seconds to locate the hidden platform. If the mouse failed to find the platform within 120 seconds, it was manually guided to the platform and allowed to remain there for 10 seconds to consolidate its spatial memory. The escape latency (time to reach the platform) was recorded as a primary indicator of spatial learning.

On post-injury days 12 and 24 after training, the platform was removed, and the mice underwent a 30-second probe trial. Mice were released from the quadrant opposite the target quadrant, and their movements were tracked using a video tracking system (EthoVision XT 16; Noldus Information Technology, Wageningen, Netherlands). Parameters recorded included the percentage of time spent and swimming distance in the target quadrant, which helped evaluate reference memory retention.

### Sucrose preference test

On post-injury day 7, the sucrose preference test (SPT) was performed (*n* = 6 per group) to measure anhedonia, a typical symptom of depression-like behavior following TBI. According to a previously documented method (Yao et al., 2022), mice were housed individually and provided with two identical bottles containing a sucrose solution (1%) and drinking water for a duration of 12 hours before the formal test. After 24 hours of food and water deprivation, the SPT was conducted for 2 days as follows: each mouse was allowed unrestricted access to the 1% sucrose solution and drinking water for 12 hours, with the positions of the two bottles switched during the second 12-hour period. Before and after the SPT, the weights of the two bottles were recorded, and sucrose preference was calculated as follows: Sucrose preference (%) = (sucrose consumption/total consumption) × 100%.

### Forced swimming test

The forced swimming test (FST) was conducted on post-injury day 7 (*n* = 6 per group) in accordance with a previous study (Yao et al., 2022). Each mouse was placed into a Perspex cylinder (height: 30 cm, diameter: 18 cm) containing water (depth: 16 cm, 22 ± 0.5°C) for 6 minutes. The duration of immobility and swimming was recorded, and the entire experiment was captured using the SMART 3.0 imaging system (Panlab, Germany).

### Statistical analysis

All experimental data were presented as mean ± standard deviation (SD). The data were analyzed using SPSS 22.0 (IBM Corporation, Armonk, NY, USA). The latency time in the MWM was analyzed by repeated-measures analysis of variance. The data were analyzed using one-way analysis of variance followed by Tukey’s *post hoc* test and independent samples *t-*test. A *P*-value < 0.05 was considered statistically significant.

## Results

### Semaglutide improves neurological functions after traumatic brain injury

**Figure 1:**
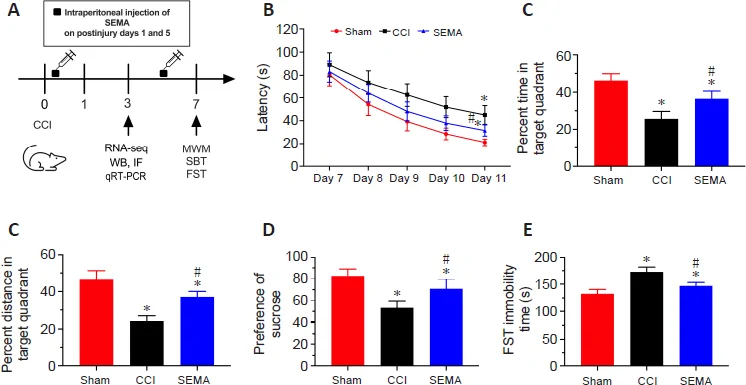
SEMA treatment improves neurological functions after traumatic brain injury. (A) Experimental designs and animal groups with different treatments and neurological function assessments. (B) Quantification of escape latency on post-injury days 7–11 (repeated-measures analysis of variance). (C, D) Quantification of the percentage of time and distance. (E) Quantification of sucrose preference on post-injury day 7. (F) Quantification of immobility time in the FST on post-injury day 7. The data are presented as the mean ± SD (*n* = 6; C–F: one-way analysis of variance followed by Tukey’s multiple comparison test). *P < 0.05, *vs*. sham group; #*P* < 0.05, *vs*. CCI group. CCI: Controlled cortical impact; FST: forced swim test; qRT-PCR: quantitative reverse transcription-polymerase chain reaction; SEMA: semaglutide.

To assess the roles of SEMA in neurobehavioral functions following CCI, the MWM, SPT, and FST were performed, and the experimental design and timeline of these tests are summarized in **Figure 1A**. The MWM test was used to assess spatial learning and reference memory, and training sessions were conducted daily for 5 consecutive days (on post-injury days 7–11). The results showed a significant reduction in escape latency over the training period in all groups (*P* < 0.05), reflecting the acquisition of spatial learning. However, in comparison with the sham-operated controls, CCI-injured mice without SEMA treatment showed significant prolongation in escape latency (*P* < 0.05), indicating impaired spatial learning ability. In contrast, SEMA-treated CCI mice showed a marked improvement in learning, with escape latency significantly lower than those of untreated CCI controls (*P* < 0.05; **Figure 1B**). At 24 hours after the final MWM training (post-injury day 12), the probe trial was performed to evaluate reference memory retention. Consistent with the training results, untreated CCI mice showed significant deficits in reference memory, and the results showed significant reductions in the goal quadrant percentages of both the time and distance traveled in comparison with sham controls (*P* < 0.05). Notably, SEMA treatment effectively restored these memory deficits: both the goal quadrant percentage of time spent and distance traveled increased significantly in comparison with those in untreated CCI mice (*P* < 0.05; **Figure 1C** and **D**).

As described previously, both SPT and FST were conducted 7 days post-TBI to detect depressive-like behaviors. The post-TBI sucrose preference rates for the mice in the CCI group were significantly lower than those in the sham group (*P* < 0.05), indicating the occurrence of depressive-like symptoms (anhedonia) in these mice after TBI. In the FST, the data revealed that the immobilization time of CCI mice was significantly greater than that of the sham controls (*P* < 0.05), indicating the development of behavioral despair in the CCI mice. However, SEMA treatment significantly increased the sucrose preference rate and reduced the immobility time (*P* < 0.05) in comparison with the corresponding values in the CCI controls (**Figure 1E** and **F**). In conclusion, these findings collectively suggest that SEMA administration contributes to recovery of neurological functions after TBI.

### Semaglutide attenuates apoptosis and promotes neuronal survival after traumatic brain injury

**Figure 2:**
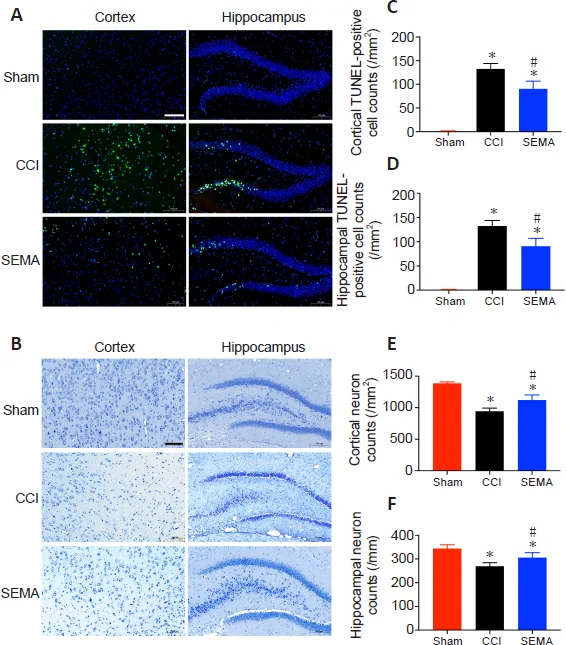
SEMA treatment reduces apoptosis and promotes neuronal survival in the injured cortex and ipsilateral hippocampus after brain injury. (A) Representative immunofluorescence images of TUNEL^+^ cells (scale bar: 50 μm; green fluorescence). (B) Representative immunofluorescence images of Nissl^+^ cells (Scale bar: 100 μm for the cortex; 200 μm for the hippocampus; blue staining). (C, D) Quantification of the number of TUNEL^+^ cells in the injured cortex and ipsilateral hippocampus on post-injury day 3. (E, F) Quantification of the number of Nissl^+^ cells in the injured cortex and ipsilateral hippocampus on post-injury day 7. The data are presented as the mean ± SD (*n* = 6; one-way analysis of variance followed by Tukey’s multiple comparison test). *P < 0.05, *vs*. sham group; #*P* < 0.05, *vs*. CCI group. CCI: Controlled cortex impact; SEMA: semaglutide; TUNEL: TdT-mediated dUTP Nick-End Labeling.

To estimate the effects of SEMA in preserving neurons against injury after TBI, neuronal survival (Nissl staining) and cell death (TUNEL assay) were assessed at post-injury days 3 and 7, respectively (**Figure 2A** and **B**). The brain regions that are injured during trauma as well as those that are particularly sensitive to TBI, such as the prefrontal and temporal regions in the perilesional cortex and the hippocampus, which also play essential roles in cognitive processing and memory functions (spatial memory), were analyzed in the brains of mice with CCI injury. TUNEL results showed that the number of TUNEL^+^ cells was significantly greater in the ipsilateral cortex and hippocampus after TBI. In contrast, SEMA treatment inhibited apoptosis and significantly reduced the number of TUNEL^+^ cells in both the ipsilateral cortex and hippocampus (*P* < 0.05; **Figure 2C** and **D**). Our previous work showed that the continuous loss of neurons around the lesion post-TBI and the reduction in the number of neurons reach their maximum values on post-injury day 7. In this study, the Nissl staining results showed significant (*P* < 0.05) reduction in the number of Nissl^+^ cells in the cortex and hippocampus areas of the brain on post-injury day 7. Application of SEMA reversed this condition by significantly increasing the number of Nissl^+^ cells in the ipsilateral cortex and hippocampus (*P* < 0.05; **Figure 2E** and **F**). Together, these findings suggest that SEMA prevented early-onset injury-induced neurological damage and promoted neuronal survival.

### Semaglutide decreases blood–brain barrier permeability and attenuates post-injury cerebral edema

**Figure 3:**
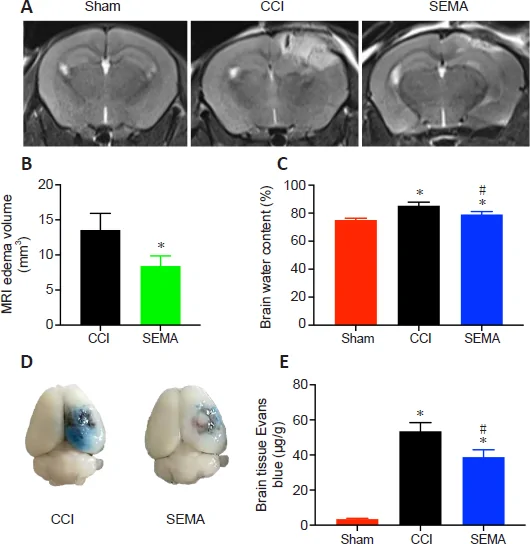
SEMA decreases BBB permeability and attenuates post-TBI brain edema. (A) Representative images of T2-weighted MRI. (B, C) Quantification of edema volume around the injury site and brain water content in the ipsilateral hemisphere on post-injury day 3. (D) Representative brain images showing Evans blue. (E) Quantification of Evans blue extravasation in the ipsilateral hemisphere on post-injury day 3. The data are presented as the mean ± SD (*n* = 6; B: independent samples *t*-test; C, E: one-way analysis of variance followed by Tukey’s multiple comparison test). *P < 0.05, *vs.* sham group; #*P* < 0.05, *vs*. CCI group. BBB: Blood–brain barrier; CCI: controlled cortical impact; SEMA: semaglutide; TBI: traumatic brain injury.

Brain edema, which is one of the most serious secondary injuries following brain injury and can lead to severe increase in intracranial pressure, peaks at approximately 1–3 days after TBI. To evaluate the effects of SEMA on brain edema, wet/dry weight ratio measurements and MRI volumetry were used to quantify edema in the injured hemisphere on post-injury day 3 (*n* = 6 per group). The results showed that the brain water content and edema volume were significantly elevated (*P* < 0.05) after CCI than in the sham controls. In contrast, SEMA treatment significantly attenuated both parameters in comparison with CCI controls (*P* < 0.05; **Figure 3A–C**). These results indicate the efficacy of SEMA in reducing brain edema after TBI.

Disruption of the BBB is one of the important mechanisms for post-traumatic vasogenic edema. To investigate the detailed mechanism underlying the effects of SEMA in alleviating post-traumatic edema, BBB permeability in the injured brain hemisphere on post-injury 3 day was evaluated by EB extravasation. As depicted in **Figure 3D** and **E**, in comparison with the findings in the sham group, EB extravasation was significantly greater in the ipsilateral brain hemispheres (*P* < 0.05) in the CCI group, which indicate greater permeability of the BBB. However, SEMA treatment significantly reduced the extravasation of EB from the injury-side brain hemisphere (*P* < 0.05). Thus, the results of the EB test showed that SEMA could repair BBB, reduce its permeability, and thereby inhibit cerebral edema.

### Semaglutide preserves blood–brain barrier integrity after traumatic brain injury

**Figure 4:**
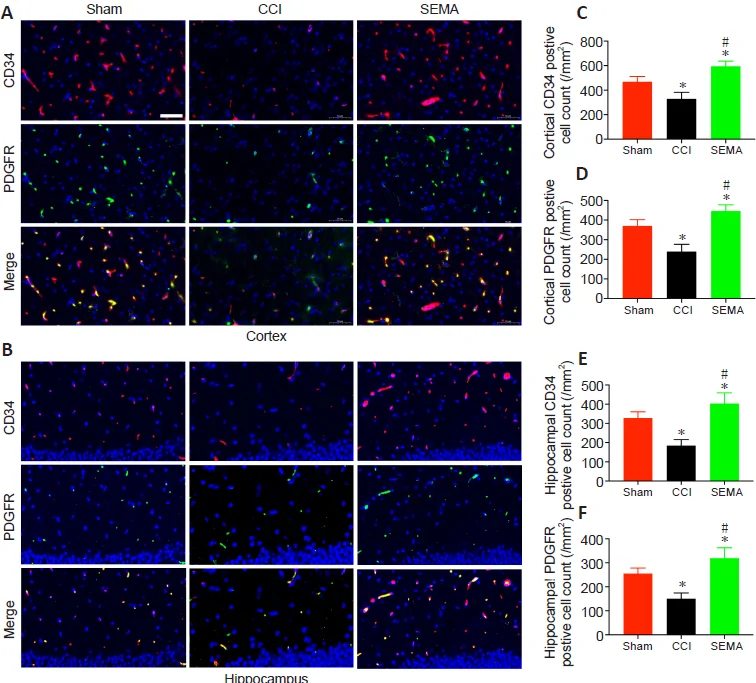
SEMA preserves blood–brain barrier integrity after brain injury. (A, B) Representative images of PDGFR^+^ (green fluorescence) and CD34^+^ (red fluorescence) cells in the injured cortex and ipsilateral hippocampus at 3 days (Scale bar: 50 μm). (C–F) Quantification of PDGFR^+^ and CD34^+^ cells in the injured cortex and ipsilateral hippocampus. The data are presented as the mean ± SD (*n* = 6; one-way analysis of variance followed by Tukey’s multiple comparison test). *P < 0.05, *vs*. sham group; #*P* < 0.05, *vs.* CCI group. CCI: Controlled cortical impact; PDGFR: platelet-derived growth factor receptor; SEMA: semaglutide; ZO-1: Zonula occludens-1.

Endothelial cells, pericytes, and the endfeet of astrocytes constitute the main components of the BBB, and their dysfunction or injury can impair BBB integrity after TBI. To assess the effects of SEMA on BBB structure, immunohistochemical staining of CD34 and PDGFRβ was performed to quantify the number of endothelial cells and pericytes in the ipsilateral cortex and hippocampal regions at 3 days after TBI (**Figure 4A** and **B**). The results revealed significant reductions in both CD34^+^ endothelial cell density and PDGFRβ^+^ pericytes in the perilesional cortical and hippocampal tissues in comparison with the sham group (*P* < 0.05). In contrast, SEMA treatment protected the BBB components and significantly increased the number of endothelial cells and pericytes (*P* < 0.05; **Figure 4C–F**). The results indicated that one of the mechanisms by which SEMA exerted neuroprotective effects was by maintaining the integrity of BBB core structures such as endothelial cells and pericytes in the injured regions after CCI, thereby laying the foundation for BBB recovery and restoration in the injured cerebrovascular regions and BBB.

**Figure 5:**
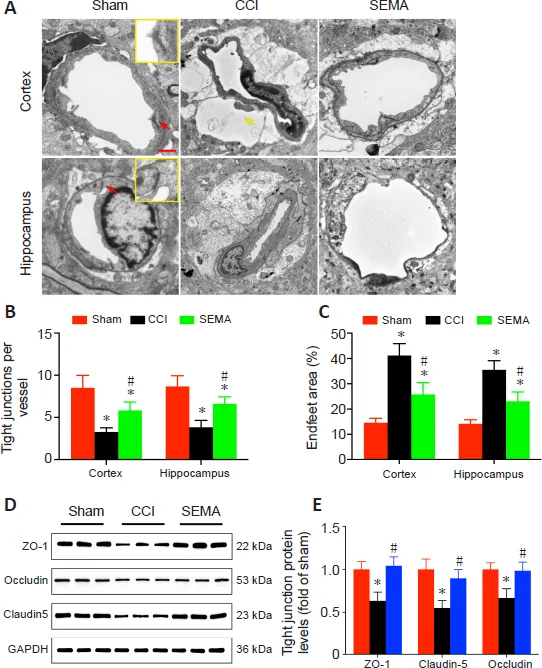
The ultrastructural alterations of the blood–brain barrier (BBB) after brain injury. (A) Representative images of TEM showing the ultrastructure of BBB (scale bar: 2 μm). (B, C) Quantification of the number of tight junctions (red arrows) and the endfoot area (yellow arrow). (D) Representative images of western blot showing ZO-1, claudin-5 and occludin. (E) Quantification of the expressions of ZO-1, claudin-5, and occludin. The relative band density was normalized to that of GAPDH, and the percent expression compared to that of sham controls was calculated for each sample. The data are presented as the mean ± SD (*n* = 6; one-way analysis of variance followed by Tukey’s multiple comparison test). *P < 0.05, *vs*. sham group; #*P* < 0.05, *vs.* CCI group. CCI: Controlled cortical impact; GAPDH: glyceraldehyde-3-phosphate dehydrogenase; SEMA: semaglutide; TEM: transmission electron microscope; ZO-1: Zonula occludens-1.

TBI-induced BBB dysfunction is accompanied by ultrastructural damage (e.g., tight junction loss, astrocytic endfoot edema). To characterize the effects of SEMA on the BBB ultrastructure, transmission electron microscopy (TEM) was used to observe the microvascular features in the peri-injury cortex (3 days post-CCI). TEM demonstrated ultrastructural alterations in the BBB post-TBI, including fewer tight junctions (*P* < 0.05) and astrocytic endfoot swelling. In contrast, SEMA attenuated these changes, increased the number of tight junctions and reduced edema in astrocyte endfeet (**Figure 5A–C**).

In addition, to characterize the regulatory effects of SEMA on BBB-related proteins, western blot analysis was performed to detect the expression levels of tight junction proteins, including ZO-1, occludin, and claudin-5. The results showed that the expression levels of these proteins reduced significantly after injury, whereas SEMA treatment significantly upregulated their expression levels (*P* < 0.05; **Figure 5D** and **E**), indicating that SEMA improved BBB integrity at protein level as well.

### The cAMP-response element binding protein and phosphoinositide 3-kinase/protein kinase B signal pathways are involved in semaglutide-mediated neuroprotective effects after traumatic brain injury

**Figure 6:**
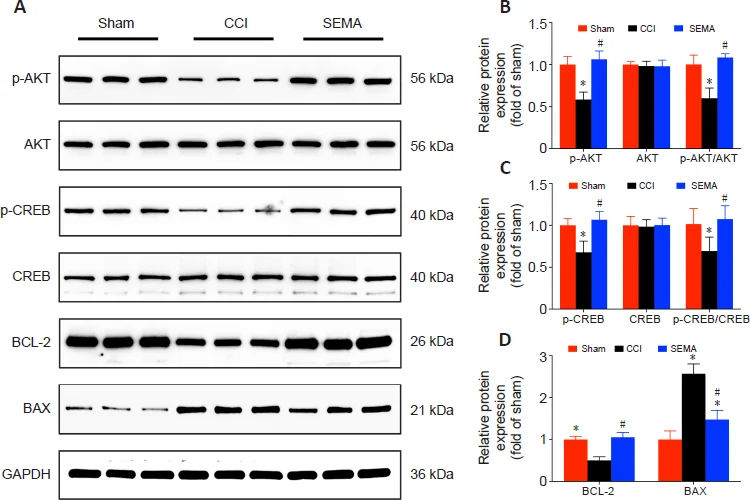
SEMA promotes neuronal survival by activating AKT/CREB pathway after brain injury. (A) Representative western blot images showing expressions of AKT, CREB, BCL-2, and BAX; (B–D) Quantification of AKT, CREB, BCL-2, and BAX. The relative band density was measured using ImageJ (1.49 V) and normalized to that of GAPDH, with percent expression compared to sham controls calculated for each sample. The data are presented as the mean ± SD (*n* = 6; one-way analysis of variance followed by Tukey’s multiple comparison test). *P < 0.05, *vs*. sham group; #*P* < 0.05, *vs.* CCI group. AKT: Protein kinase B; BAX: Bcl-2 associated X protein; BCL-2: B-cell lymphoma 2; CCI: controlled cortical impact; CREB: cAMP-response element binding protein; GAPDH: glyceraldehyde-3-phosphate dehydrogenase; SEMA: semaglutide.

The neuroprotective effects of GLP1-RAs have been previously shown to involve promotion of the phosphoinositide 3-kinase (PI3K)/AKT and CREB pathways, which regulate cell apoptosis and survival. Therefore, to evaluate the effects of SEMA on these signaling pathways, we detected the presence of phospho-/total-AKT (p-AKT/AKT), phospho-/total-CREB (p-CREB/CREB) and the proteins of cell apoptosis (Bcl-2 and Bax) in the injured hemisphere on post-injury day 3. Western blot results showed that expression of p-AKT and p-CREB increased significantly in mice after SEMA treatment than in those from the CCI control group. Accordingly, the p-AKT/AKT ratio as well as the p-CREB/CREB ratio also increased after SEMA treatment (*P* < 0.05; **Figure 6**). Meanwhile, in comparison with the CCI control mice, mice from the SEMA group showed increased expression of Bcl-2 (an anti-apoptotic protein) and decreased expression of Bax (a pro-apoptotic protein). These results suggested that the PI3K/AKT/CREB pathway may be involved in the neuroprotective effects of SEMA after TBI.

### Semaglutide suppresses the activation of proinflammatory microglia and astrocytes in the perilesional region following traumatic brain injury

**Figure 7:**
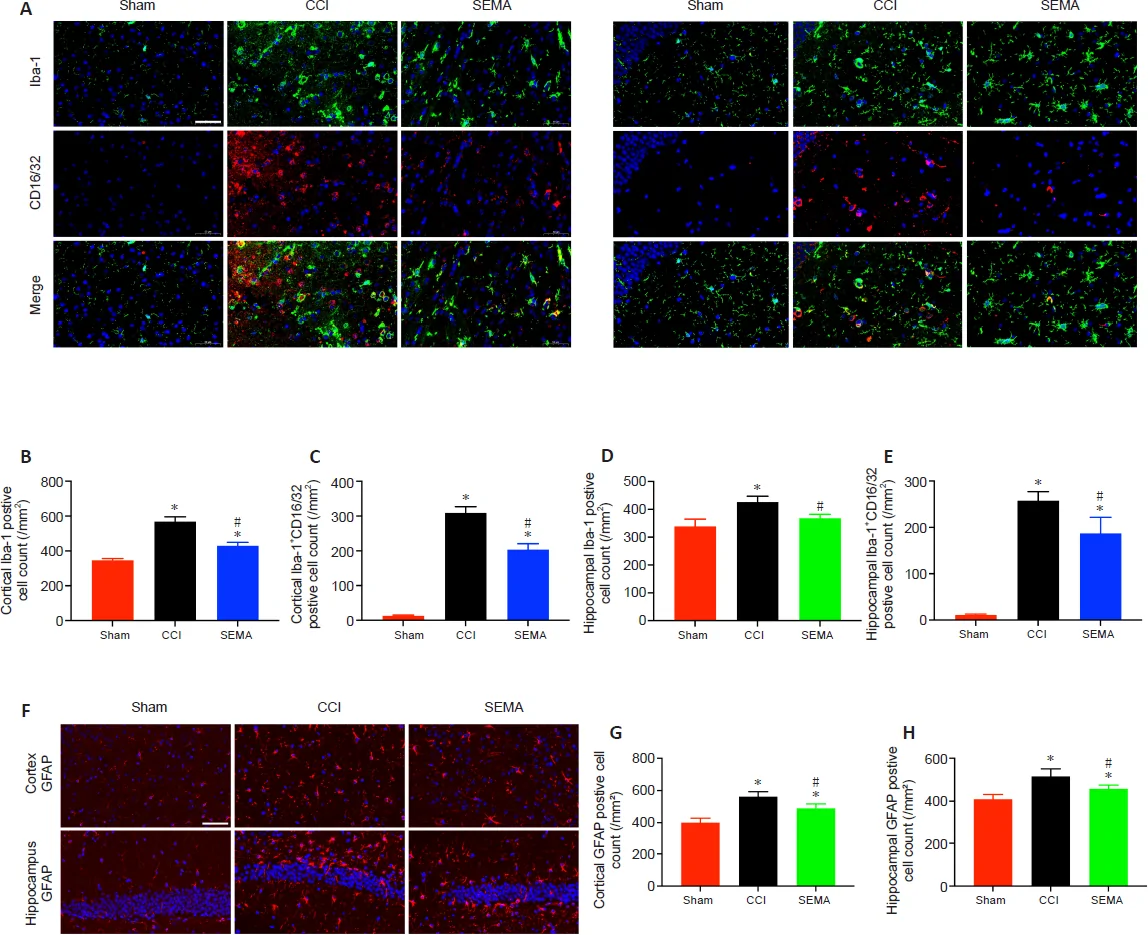
SEMA inhibits the activations of microglia and astrocytes in the injured cortex and ipsilateral hippocampus after brain injury. (A) Representative immunofluorescence images of Iba1^+^ (green fluorescence) and CD16^+^/32^+^ (red fluorescence) cells (scale bar: 50 μm). (B–E) Quantification of Iba1^+^ and CD16^+^/32^+^ cells. (F) Representative immunofluorescence images of GFAP^+^ cells (red fluorescence; scale bar: 50 μm). (G, H) Quantification of GFAP^+^ cells. The data are presented as the mean ± SD (*n* = 6; one-way analysis of variance followed by Tukey’s multiple comparison test). *P < 0.05, *vs.* sham group; #*P* < 0.05, *vs.* CCI group. CCI: Controlled cortical impact; GFAP: glial fibrillary acidic protein; Iba-1; ionized calcium-binding adapter molecule 1; SEMA: semaglutide.

Microglia and astrocytes are the main innate immune cells in the brain. After TBI, they are activated by injury-related molecular patterns (DAMPs), thereby initiating an inflammatory response. The inflammatory response and inflammatory mediators can damage the BBB and promote the infiltration of peripheral immune cells, further exacerbating the inflammatory response and aggravating secondary damage. To determine the effects of SEMA on neuroinflammation, we used immunofluorescence technology to quantitatively analyze the activated microglia and astrocytes in the damaged cortex and hippocampus on post-injury day 3. The results revealed that CCI induced a significant increase in the numbers of proinflammatory microglia (Iba1^+^ CD16/CD32^+^) and reactive astrocytes (GFAP^+^) in the ipsilateral cortex and hippocampus in comparison with sham controls (*P* < 0.05). In contrast, SEMA treatment substantially attenuated this response and reduced the number of Iba1^+^ CD16/CD32^+^ microglia and GFAP^+^ astrocytes in this region in comparison with the CCI group (*P* < 0.05; **Figure 7**). These findings suggest that SEMA mitigated TBI-induced glial hyperactivation, a critical step in dampening neuroinflammatory pathways and preserving central nervous system homeostasis. These findings also indicate that SEMA could alleviate the excessive activation of glial cells caused by TBI, which is a crucial step in inhibiting the inflammatory pathways and alleviating secondary brain injury.

### Transcriptomic profiling and differentially expressed genes in the mouse cortex after traumatic brain injury and semaglutide treatment

**Figure 8:**
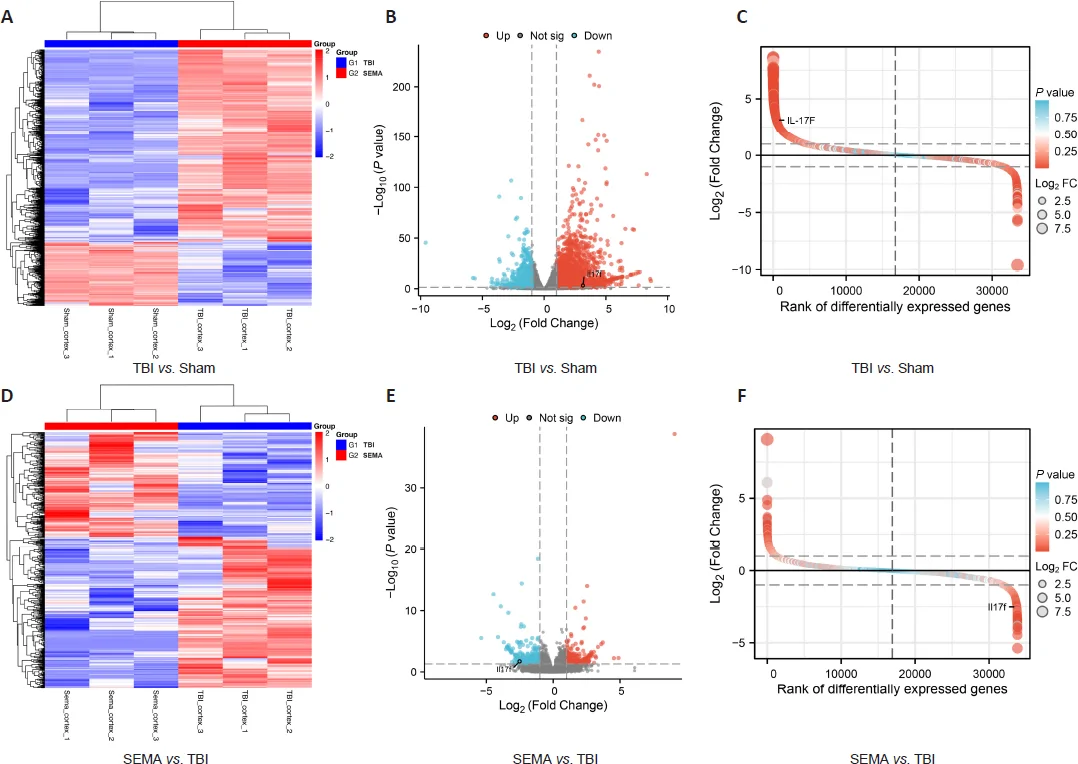
Distribution of differentially expressed genes in the sham, TBI, and SEMA groups (*n* = 3). (A) Heatmap displaying the hierarchical clustering of DEGs in the cortex (TBI *vs*. sham). (B) Volcano plot showing DEGs in the cortex (TBI *vs.* sham; red: upregulation; blue: downregulation). (C) Gene sequencing map illustrating the expression level of IL-17F in the cortex (TBI *vs.* sham). (D) Heatmap displaying the hierarchical clustering of DEGs in the cortex (SEMA *vs*. TBI). (E) Volcano plot showing DEGs in the cortex (SEMA *vs.* TBI; red: upregulation; blue: downregulation). (F) Gene sequencing map illustrating the expression level of IL-17F in the cortex (SEMA *vs.* TBI). Upregulated and downregulated DEGs are highlighted in red and blue, respectively. The color intensity and size of the plots indicate the degree of the *P* value and log_2_ (fold change). DEGs: Differentially expressed genes; Il-17F: interleukin-17F; SEMA: semaglutide; TBI: traumatic brain injury.

Nine libraries were assigned to the sham, TBI, and SEMA groups, respectively, and subjected to sequencing using the Illumina HiSeq platform. Sequencing results revealed that a total of 3419 DEGs (|log_2_(fold change)|>1, *P* < 0.05) were detected by comparison of the sequencing data from the CCI and sham groups on post-injury day 3. Among these DEGs, 2582 were upregulated and 837 were downregulated in the cortical tissue (**Figure 8A–C**). Furthermore, a total of 583 DEGs distinguished the SEMA group from the CCI group, of which 239 genes were upregulated and 344 genes were downregulated (**Figure 8D–F**). Among them, IL-17F was significantly upregulated after CCI, while SEMA treatment significantly reduced IL-17F level.

GO annotation and KEGG analysis were performed to further investigate the functions of the DEGs after CCI and SEMA treatment. The results showed significant positive regulation of innate and peripheral inflammatory responses and cytokine expressions, such as the nuclear factor (NF)-kB pathway, TNF signaling pathway, Toll-like receptor signaling pathway, Janus kinase (JAK)/STAT pathway, NLRP3, IL-1, IL-6, TNF, and IL-17, which indicated that the inflammatory response following TBI played crucial roles in the process of secondary brain injury. However, SEMA treatment induced a transcriptional signature characterized by downregulation of inflammatory processes, including lymphocyte/mononuclear cell proliferation, leukocyte activation, and Th17 cell differentiation, which are key steps in resolving neuroinflammation. Among these, IL-17 emerged as a central hub connecting peripheral immune cell infiltration (e.g., Th17 cells) and innate glial activation in the injured brain. Thus, TBI significantly upregulated the components of the IL-17 pathway, while SEMA treatment inhibited this response, consistent with the anti-inflammatory and neuroprotective properties of SEMA. These findings clearly indicate that the immune interactions mediated by IL-17 are closely linked to the pathological process of TBI. However, the specific details of the regulatory mechanism underlying the effects of SEMA on IL-17-dependent neuroinflammation require further investigation (**Additional Figure 1**).

### The interleukin-17/NOD-like receptor family pyrin domain containing 3/signal transducer and activator of transcription 3 signal pathway may be associated with the semaglutide-mediated anti-inflammatory effects after traumatic brain injury

**Figure 9:**
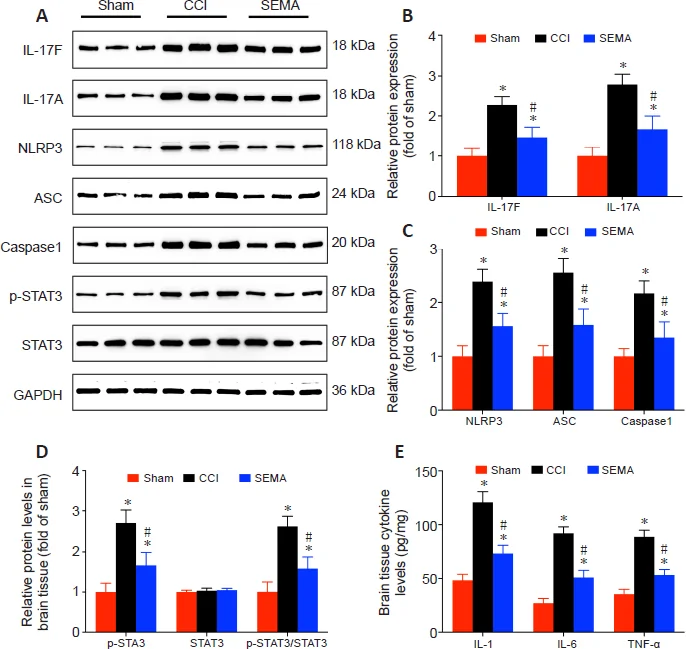
The anti-inflammatory effects of SEMA associated with Il-17/NRLP3/JAK/STAT3 pathway after traumatic brain injury. (A) Representative western blot images showing the expressions of IL-17A, IL-17F, NLRP3, ASC, caspase-1, p-STAT3, and STAT3. (B–D) Quantification of IL-17A, IL-17F, NLRP3, ASC, C-caspase-1, p-STAT3, and STAT3 in the ipsilateral hemisphere; (E) Quantification of IL-1, IL-6, and TNF-α in the ipsilateral hemisphere. Relative band density was normalized to GAPDH levels, with percent expression compared to sham controls calculated for each sample. Data are presented as mean ± SD (*n* = 6; one-way analysis of variance followed by Tukey’s multiple comparison test). *P < 0.05, *vs*. sham group; #*P* < 0.05, *vs.* CCI group. ASC: Apoptosis-associated speck-like protein containing a caspase recruitment domain; CCI: controlled cortical impact; NLRP3: NOD-like receptor pyrin domain-containing 3; SEMA: semaglutide; STAT: signal transducer and activator of transcription 3.

**Figure 10:**
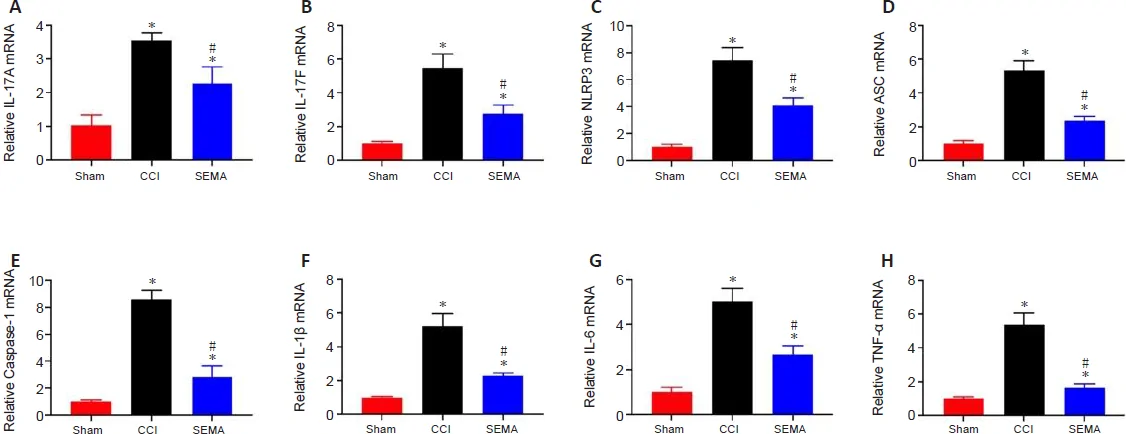
Effects of SEMA on the mRNA levels of inflammatory cytokines in brain tissue in the ipsilateral hemisphere cortex after traumatic brain injury. (A–H) Quantification of the relative mRNA levels of IL-17A, IL-17F, NLRP3, ASC, Caspase-1, IL-1β, IL-6, and TNF-α in microglia isolated from the ipsilateral cortex. The data are presented as the mean ± SD (*n* = 5; one-way analysis of variance followed by Tukey’s multiple comparison test). *P < 0.05, *vs.* sham group; #*P* < 0.05, *vs*. CCI group. ASC: Apoptosis-associated speck-like protein containing a caspase recruitment domain; CCI: controlled cortical impact; IL: interleukin-6; NLRP3: NOD-like receptor pyrin domain-containing 3; SEMA: semaglutide; TNF-α: tumor necrosis factor-alpha.

Emerging evidence highlights the critical role of the bidirectional IL-17-NLRP3 inflammasome axis for amplifying neuroinflammation, with JAK/STAT signaling driving microglial and macrophage activation following central nervous system injury. To clarify the molecular mechanisms underlying the anti-inflammatory effects of SEMA, we investigated the IL-17/NLRP3/STAT3 signaling cascade and associated proinflammatory cytokines in the injured brain. The polymerase chain reaction (PCR) and western blot results showed that the levels of IL-17A, IL-17F, NLRP3, ASC, caspase 1, and p-STAT3 as well as the p-STAT3/STAT3 ratio in the ipsilateral hemisphere significantly increased on post-injury day 3. In contrast, SEMA treatment significantly reduced the expressions of the above cytokines (*P* < 0.05; **Figure 9A–D**). In addition, ELISA results revealed that TBI significantly upregulated the expression levels of IL-1β, IL-6, and TNF-α in the ipsilateral hemisphere in comparison with sham controls. In contrast, SEMA significantly reduced the levels of these cytokines, which is consistent with its anti-inflammatory effect (*P* < 0.05; **Figure 9E**). Accordingly, PCR assays of the ipsilateral hemisphere showed that SEMA treatment significantly reduced the mRNA levels of these proinflammatory factors (*P* < 0.05; **Figure 10**).

Collectively, these results indicate that SEMA disrupted the pathogenic positive-feedback loop between the central and peripheral immune cells by suppressing the IL-17/NLRP3/STAT3 axis. This dual inhibition of upstream inflammasome activation and downstream cytokine production effectively attenuated the neuroinflammatory cascade, representing a pivotal mechanism by which SEMA mitigates secondary brain injury after traumatic insult.

## Discussion

This study explored the therapeutic effects of SEMA in a mouse CCI model, particularly its effects on neuroinflammation, BBB integrity, cerebral edema, neuronal survival, and functional recovery as well as the underlying molecular pathways. Our results showed that SEMA treatment protected the BBB, reduced brain edema, improved neuronal survival, and enhanced neurological recovery after TBI. Notably, SEMA significantly suppressed the activation of innate immune cells, including microglia and astrocytes, in the injured hemisphere, and this improvement was accompanied by a substantial downregulation of proinflammatory cytokines such as IL-1β, TNF-α, and IL-6. Mechanistically, these protective effects may be linked to the inhibition of the IL-17/NLRP3 pathway, which was indicated by reduced levels of IL-17, NLRP3, caspase-1 activation, and IL-1β, alongside modulation of the JAK/STAT3 signaling axis, which collectively attenuated neuroinflammatory cascades. These results highlight the role of SEMA in inhibiting inflammation and mitigating secondary injury by targeting the positive feedback loop between peripheral and central innate immune cells mediated by IL-17/NLRP3 following TBI.

After TBI, neuroinflammation, cerebral edema, and elevated ICP are critical and life-threatening forms of secondary injury (Thapa et al., 2021; Maas et al., 2022). These pathologies, which are driven by BBB disruption and inflammatory infiltration, exacerbate neuronal damage even after decompressive surgery. However, beyond surgical interventions, effective pharmacotherapeutic approaches targeting the underlying mechanisms of these pathologies remain limited (Halstead et al., 2019; Jha et al., 2019). Glucocorticoids, despite their potent anti-inflammatory activity, are not recommended for severe TBI due to systemic risks (Bratton et al., 2007), while mannitol only provides transient osmotic relief to reduce the ICP without addressing neuroinflammation or BBB damage, and may worsen edema in compromised regions (Tani et al., 2022). Additionally, sulfonylureas such as glibenclamide have failed to achieve clinical translation due to inconsistent results and the risk of hypoglycemia (Lin et al., 2024; Razavi et al., 2024; Sheth et al., 2024). In this context, GLP-1RAs are promising alternatives supported by the widespread expression of GLP-1R in TBI-vulnerable brain regions and their localization in neurons, astrocytes, and microglia, which can enable dual modulation of neural signaling and neuroimmune responses (Kabahizi et al., 2022; Zhang et al., 2023). Previous studies on GLP-1RAs (e.g., exendin-4) in animal TBI models have shown neuroprotective effects, including reduced neuroinflammation and apoptosis (Lv et al., 2023; Harej Hrkać et al., 2024). SEMA, a long-acting GLP-1RA, offers unique advantages: sustained pharmacokinetics (half-life up to 7 days), established safety in metabolic disorders, and a glucose-dependent hypoglycemic effect that minimizes side effects (Bliddal et al., 2024; McGowan et al., 2024; Maretty et al., 2025). Our results show that SEMA effectively reduces cerebral edema, inhibits neuronal apoptosis, promotes neuronal survival, and improves functional deficits in acute TBI, highlighting its superior translational potential in comparison with existing therapies. Unlike mannitol, it targets the mechanistic basis of edema (e.g., preserving BBB integrity by increasing CD34^+^ endothelial cell density and tight junction protein expression); unlike glucocorticoids, it avoids systemic harm; and unlike glibenclamide, it exhibits consistent preclinical efficacy without the risk of hypoglycemia. This advantage arises from the interaction of SEMA with GLP-1Rs across the neural and glial compartments, which allows it to mitigate multiple secondary injury cascades and thereby exert a broader action than single-target agents. Moreover, the long-acting profile of SEMA aligns with clinical needs for sustained protection, extending the neuroprotective framework of GLP-1RAs from neurodegenerative diseases to acute TBI (Salameh et al., 2020; Zhang et al., 2023; Meca et al., 2024; Poupon-Bejuit et al., 2024; Wang W et al., 2024; Cummings et al., 2025).

TBI initiates a pronounced neuroinflammatory response through the release of DAMPs from necrotic neural cells. Upon binding to pattern-recognition receptors on immune cells, these DAMPs activate the NLRP3 inflammasome. This activation causes cleavage of caspase-1 and maturation of IL-1β, a process that amplifies proinflammatory cytokine secretion and promotes microglial activation. These events collectively constitute the core mechanism underlying secondary brain injury, causing damage to the BBB and brain edema and mediating both acute and long-term neurological damage (Morganti-Kossmann et al., 2019; Alam et al., 2020). Previous studies have revealed the anti-inflammatory effects of SEMA in AD and ischemic stroke models (Zhang et al., 2022; Meca et al., 2024; Poupon-Bejuit et al., 2024). In AD, SEMA alleviates neuroinflammation by suppressing microglial overactivation, in addition to reducing amyloid-β cytotoxicity and tau hyperphosphorylation. In a mouse model of ischemic stroke, SEMA inhibits the activation of CD16^+^/32^+^ proinflammatory microglia and reactive astrocytes, diminishes neuroinflammation, and mitigates BBB breakdown. However, its function in regulating the neuroinflammation following TBI has not been sufficiently investigated. While the neuroprotective activity of SEMA in AD and stroke indicates translational value for TBI, critical gaps remain in understanding how it modulates the unique neuroinflammatory cascades triggered by traumatic injury. Our data demonstrated that SEMA alleviated TBI-induced cerebral edema through two complementary mechanisms: first, it suppresses M1-polarized microglial activation and restricts astrocyte overactivation; second, it restores the structural integrity of the BBB, thereby reducing BBB permeability and further mitigating cerebral edema. These observations align with the well-documented anti-inflammatory properties of GLP-1RAs and expand this understanding to TBI, where neuroinflammation and BBB impairment are the primary therapeutic priorities.

The interactions between brain-resident innate immune cells (microglia, astrocytes) and infiltrating peripheral immune cells (neutrophils, monocytes) amplify the post-injury neuroinflammation after TBI. Activated glia secrete chemokines to recruit peripheral leukocytes, which release proinflammatory mediators to exacerbate glial activation and form a reinforcing inflammatory cascade (Todd et al., 2021; Grovola et al., 2023). Within this cascade, IL-17, which is primarily secreted by Th17 cells and by infiltrating neutrophils and resident microglia at the lesion site, plays a pivotal role (Huangfu et al., 2023; Xu et al., 2023). IL-17 accumulates locally to mediate crosstalk between peripheral leukocytes and central innate immune cells through its receptor (IL-17RA/RC) expressed on glia and brain microvascular endothelial cells (Piepke et al., 2021; Zheng et al., 2025). This activation drives the production of cytokines (e.g., IL-6, TNF-α) that perpetuate immune cell recruitment and neurotoxicity and promotes a bidirectional IL-17-NLRP3 inflammasome axis to amplify inflammation (Wang et al., 2019; Sui et al., 2021; Xu et al., 2025).

While prior studies have demonstrated that SEMA exerts anti-inflammatory effects by suppressing NLRP3 activation in non-TBI contexts, they did not address whether SEMA modulated the feedback loop between innate and peripheral immunity or its regulation of IL-17. Our findings extended the research on SEMA by revealing two novel effects in TBI: first, sequencing, PCR, and western blot results showed that SEMA significantly downregulated both IL-17A and IL-17F, directly targeting the cytokine mediating immune crosstalk; second, SEMA alleviated the NLRP3-induced inflammatory response in brain tissue surrounding the lesion, which was evidenced by decreased caspase-1 activity and reduced levels of ASC and IL-1β. This aligns with its inhibitory effect on NLRP3 and contextualizes this effect within TBI’s unique inflammatory network. The significance lies in the dual regulation of SEMA: by downregulating IL-17, it disrupts the crosstalk between peripheral leukocytes and central glia, while concurrent NLRP3 inhibition blocks downstream inflammation triggered by IL-17 signaling. This dual action addresses the self-reinforcing nature of TBI neuroinflammation, a dimension unaddressed by prior studies on SEMA. Unlike previous studies focused on single inflammatory targets, our study demonstrated that SEMA targeted the interconnected IL-17-NLRP3 axis and immune cell crosstalk, providing a more comprehensive anti-inflammatory mechanism in TBI and supporting the potential of SEMA as a therapeutic agent that disrupts the core inflammatory loop driving secondary brain injury.

The STAT family of transcription factors has been reported to play important roles in the activation of microglia and macrophages (Lyu et al., 2021). In an experimental retinopathy mouse model, STAT3 was found to be the key factor involved in IL-17-induced microglia activation and retinal vascular injury (Zhou et al., 2021). Our results showed that SEMA treatment significantly reduced the levels of phosphorylated STAT3, indicating that SEMA treatment blocked the positive feedback loop between IL-17/NLRP3 and STAT3 activation, which perpetuates microglial M1 polarization and proinflammatory cytokine release. This dual modulation of IL-17/NLRP3 and JAK/STAT3 pathways underscores the capacity of SEMA to break the self-reinforcing cycle between peripheral and innate immune cells, a novel mechanism distinct from conventional TBI therapies.

Experimental studies have proven that IL-17 exacerbates neuronal apoptosis in mice after stroke and TBI by downregulating AKT and CREB phosphorylation, reducing Bcl-2 expression, and upregulating Bax while elevating the Bax/Bcl-2 ratio (Chen et al., 2023). Conversely, in seizure and chronic epilepsy models, SEMA has been reported to upregulate Bcl-2 and suppress Bax and inhibit apoptosis, thereby restoring neuronal survival (Chang et al., 2020; Wang et al., 2021). While the role of IL-17 in apoptotic signaling is well-established, the effects of SEMA on apoptosis-related pathways in TBI remain unclear. Our findings demonstrate that SEMA treatment restores the balance of apoptosis-regulating proteins, evidenced by decreased Bax/Bcl-2 ratios and reduced apoptotic cell counts in the injured brain. This anti-apoptotic effect is likely mediated by the activation of the IL-17/Akt/CREB signaling axis, which may counteract IL-17-induced pro-apoptotic cascades. By modulating this pathway, SEMA promotes neuronal survival, an effect consistent with its neuroprotective roles observed in other neurological conditions. These results bridge the gap between the known anti-apoptotic properties of SEMA in epilepsy models and its novel role in TBI, highlighting the IL-17/Akt/CREB pathway as a potential therapeutic target for mitigating post-traumatic neuronal loss.

## Limitations

This study has several limitations. First, the single-dose regimen and short-term outcomes may not reflect chronic TBI pathology or long-term therapeutic effects. The sample size used for RNA-Seq was indeed relatively small. In our subsequent study, we aim to increase the sample size and conduct in-depth studies on the effects of different doses of SEMA on TBI at different time points. Second, the CCI model primarily mimics focal injury; thus, our findings did not address diffuse axonal injury mechanisms. Third, we only detected the overall expression level of IL-17 in the brain tissue around the traumatic foci after SEMA treatment, without further identifying the specific immune cell types targeted. In future studies, we will perform in-depth investigations to identify the immune cell types modulated by SEMA to regulate IL-17 expression, as well as the signaling pathways involved in mediating the crosstalk between the peripheral and central immune systems. In addition, male mice were used in this study. In our next study, we will further investigate the effects of sex and sex hormones on the SEMA-induced neuroprotective effects. Finally, the translational relevance of these findings requires validation in higher-order species and clinical cohorts.

## Conclusions

In summary, this study demonstrated that SEMA exhibits neuroprotective effects after TBI by alleviating neuroinflammation, preserving BBB integrity, reducing brain edema, and promoting neuronal survival. Mechanistically, SEMA disrupted the pathogenic IL-17/NLRP3 and JAK/STAT3 signaling, effectively inhibiting the self-reinforcing inflammatory loop between peripheral immune cells and central glial activation. Additionally, SEMA counteracted IL-17-induced apoptosis by enhancing AKT/CREB signaling to upregulate Bcl-2 while suppressing Bax expression. Additionally, SEMA mitigated IL-17-induced neuronal apoptosis by enhancing AKT/CREB signaling, a key pro-survival pathway that promoted the upregulation of Bcl-2 and concurrently suppressed the expression of Bax. These findings highlight the therapeutic potential of SEMA for TBI through dual anti-inflammatory and neuroprotective mechanisms, positioning it as a promising candidate for clinical translation. The study underscores the therapeutic potential of repurposing metabolic agents for TBI through mechanistic synergy across immune, vascular, and neuronal repair pathways.

## Additional file:

***Additional Figure 1:*** *Functional analyses of DEGs by GO, KEGG, and PPI.*

Additional Figure 1 Functional analyses of DEGs by GO, KEGG, and PPI.
(A, B) KEGG enrichment and GO analysis of DEGs in the cortex (TBI *vs*. sham). (C, D) KEGG enrichment and GO analysis of DEGs in the cortex (SEMA *vs*. TBI); DEGs were determined based on absolute log2 (fold change) values exceeding 1 and *P* < 0.05. The TBI and DEX groups consisted of three individuals each. DEGs: Differentially expressed genes; GO: gene ontology; KEGG: Kyto Encyclopedia of Genes and Genomes; PPI: protein-protein interaction; TBI: traumatic brain injury.

## Data Availability

*All relevant data are within the paper and its Additional files.*
